# Microbiota of the Rearing Water of *Penaeus stylirostris* Larvae Influenced by Lagoon Seawater and Specific Key Microbial Lineages of Larval Stage and Survival

**DOI:** 10.1128/spectrum.04241-22

**Published:** 2022-11-23

**Authors:** Nolwenn Callac, Viviane Boulo, Carolane Giraud, Maxime Beauvais, Dominique Ansquer, Valentine Ballan, Jean-René Maillez, Nelly Wabete, Dominique Pham

**Affiliations:** a Ifremer, IRD, Université de la Nouvelle-Calédonie, Université de La Réunion, CNRS, UMR 9220 ENTROPIE, Nouméa, New Caledonia; b Institut des Sciences Exactes et Appliquées (ISEA), University of New Caledonia, Nouméa, New Caledonia; Brigham Young University

**Keywords:** active microbiota, rearing water, core microbiome, specific microbiome, larvae, shrimp

## Abstract

Aquacultured animals are reared in water, where they interact with microorganisms which can be involved in their development, immunity, and disease. It is therefore interesting to study the rearing water microbiota, especially in the hatcheries of the Pacific blue shrimp Penaeus stylirostris, where larval mass mortalities occur. In this study, using HiSeq sequencing of the V4 region of the 16S rRNA molecule coupled with zootechnical and chemical analyses, we investigated whether any microbial lineages could be associated with certain mortality rates at a given larval stage. Our results indicate that the active microbiota of the rearing water was highly dynamic throughout the rearing process, with distinct communities influenced by progressive water eutrophication, larval stage, and survival rate. Our data also highlighted the role of the lagoon seawater on the rearing water microbiome, as many operational taxonomic units (OTUs) specific to a given larval stage and survival rate were detected in the primary reservoir which contained the lagoon water. We also identified biomarkers specific to water eutrophication, with *Alteromonadaceae*, *Vibrionaceae*, and *Methylophilaceae*, respectively, linked to increases in ammonia, nitrogen, and soluble reactive phosphate, or to increases in colored dissolved organic matter in the rearing water; other biomarkers were specific to certain larval stages and survival rates. Indeed, the *Marinobacteraceae* were specific to the Nauplii, and the *Thalassospiraceae* and *Saprospiraceae* to the Zoea Good condition; when mortality occurred, the *Litoricolaceae* were specific to the Zoea Bad, *Microbacteraceae* to the Mysis Bad, and *Methylophilaceae* to the Mysis Worst condition. Thus, these biomarkers might be used as potential early warning sentinels in water storage to infer the evolution of larval rearing to improve shrimp larval rearing.

**IMPORTANCE** In New Caledonia, rearing of P. stylirostris is one of the main economic activities; unfortunately, mass larval mortalities cause important production decreases, involving major economic losses for the farmers and the Territory. This phenomenon, which has occurred at any larval stage over the past decade, is poorly understood. The significance of our research is in the identification of biomarkers specific to larval stage and survival rate, with some of these biomarkers being already present in the lagoon water. This enhances the role of the lagoon on the active microbiota of the rearing water at various larval stages and survival rates. Together, our results help us understand which active microbial communities are present in the rearing water according to larval stage and health. This might lead to broader impacts on hatcheries by helping to develop useful tools for using the water—lagoon, reservoir, or rearing—to test for the presence of these biomarkers as an early monitoring strategy.

## INTRODUCTION

Aquatic animals are influenced by the physical, chemical, and microbiological composition of their surrounding water, with microorganisms playing the greatest role. Indeed, they are involved in various biogeochemical cycles, such as the carbon and nitrogen cycles, through organic matter degradation, remineralization, and organic matter turnover (1–3). In addition, microorganisms are also involved in animal development, physiology fitness, and immunity, as some microorganisms may help protect against diseases or, inversely, be pathogenic (4–7). Aquacultured animals are reared in water and are therefore continuously surrounded by the water microbiota, with which they share close relationships (8–11). Several studies have shown that microbial exchanges occur between the water and various tissues of reared aquatic animals such as the gills or intestines. Thus, the host can enrich its own microbiota by selecting microorganisms from its environment (12–14). It has also been proven that the microbiome of the larvae, especially for fish such as cod or tilapia, shared more common taxa with the surrounding rearing water than with the feed products (12, 15, 16). Many studies have argued the importance of the microbiota of the rearing water for both the health of the animal and the modulation of its microbiome during all life cycle stages (17, 18). Research on the health of reared aquatic animals or the evolution of their rearing water usually focuses on the most abundant taxa. However, for many macroorganisms, such as humans or aquatic organisms (corals or fish), it has been established that, except for a conserved core microbiome, the overall microbiota changes in response to environmental disturbances while the rare taxa are cornerstone microorganisms which shape the community structure (19–22). In addition, more and more studies have proven that the low-abundance taxa have a crucial role in the ecosystem, playing substantial roles in biogeochemical cycles and often driving concealed functions of the microbiome (22, 23).

In New Caledonia, the Pacific blue shrimp Penaeus stylirostris is farmed, and its semi-intensive production achieved up to 1,444 tons in 2019. Around 55% of this production is exported to the rest of the world, making this sector the second biggest economic source for the territory after the nickel industry (https://www.agence-rurale.nc/filieres/peche-et-aquaculture/crevettes/) (24, 25). In 2019, 128 million post-larvae were produced, compared to 167 million in 2005. This dramatic drop is due to the huge mortality issues occurring at all larval stages which hatcheries have been facing for the past decade. This phenomenon is still poorly understood, and the causes are still unidentified (24). Water quality has been considered as a possible cause, but the discrepancy in larval survival during the same rearing cycle in the same hatchery eliminates this hypothesis. Bacterial infection has often been suspected, but an analysis conducted by the Neo-Caledonian Network of Shrimp Epidemiological Vigilance (REC-DAVAR) revealed no evidence of larval septicemia. A multifactorial cause seems to prompt mass larval mortality. Our hypothesis is that a shift in the active microbial diversity of the rearing water could lead to microbial dysbiosis in both water and larvae, which could then hamper the larvae and lead to the mass mortality observed. To address whether the microbiome of the rearing water could be linked to lower larval survival rates, we especially investigated whether any microbial lineages or families were associated with certain mortality rates at given larval stages and whether the composition of the rearing seawater had any effect. Because antibiotics are used for prophylactic reasons under veterinary advice during shrimp larval rearing in New Caledonian hatcheries, we also investigated the effect of the addition or absence of erythromycin on the rearing water microbiota and larval survival. Thus, we followed the evolution of the dissolved organic matter, dissolved total nitrogen, reactive phosphorus, and active microbial diversity from the storage water to the rearing tanks during the first 10 days of larval rearing. A core microbiome of all the rearing waters was found and most of these operational taxonomic units (OTUs) were previously present in the water storage, highlighting the huge influence of the lagoon seawater on the larval rearing. Several biomarkers at the family level were specific to a given larval stage and survival rate or specific to the rearing water eutrophication. Furthermore, in the context of our study, we highlighted potential bacterial lineages present during early rearing that could either be putative larvae pathogen or potential indicators of upcoming and progressive larval mortality.

## RESULTS

### Time-series analyses of the chemical parameters and larval development.

**FIG 1:**
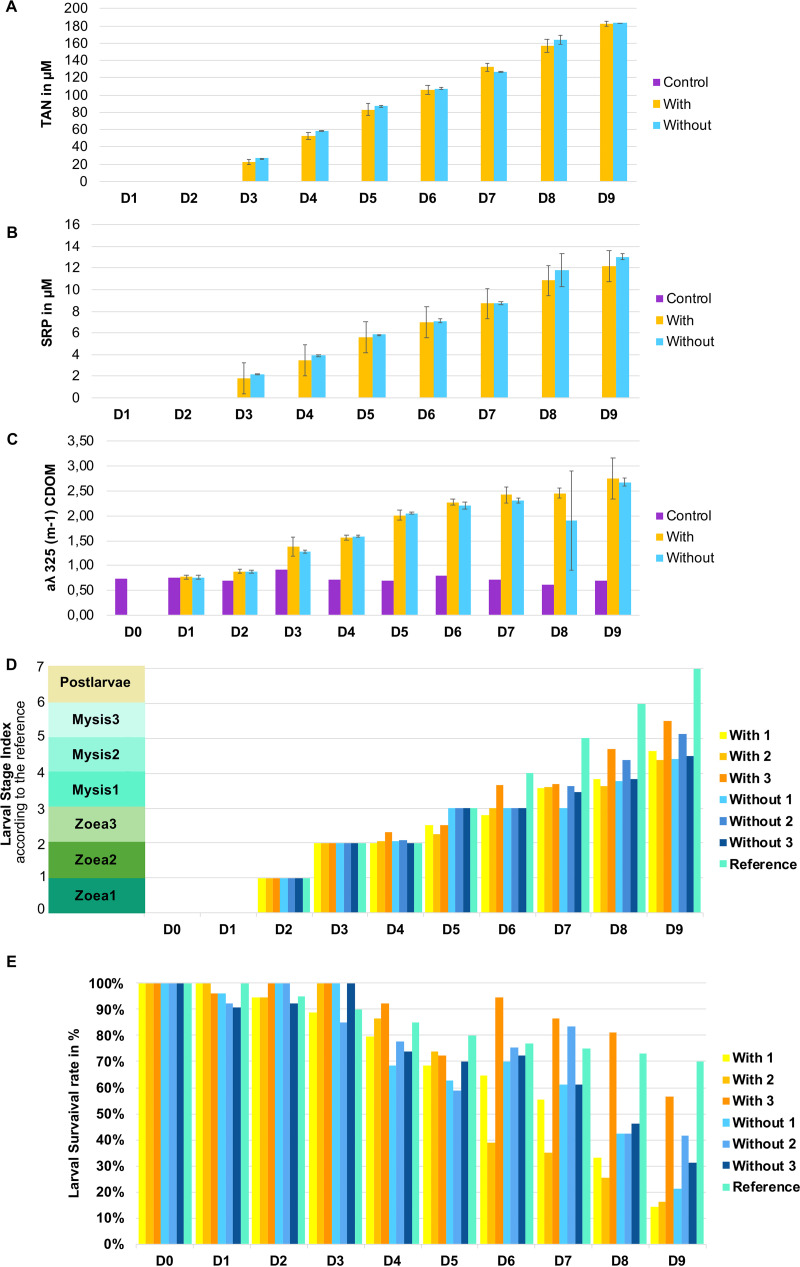
Evolution of hydrochemical values, larval survival rates, and development according to the days of the rearing period. Evolution of (A) total ammonia nitrogen (TAN), (B) soluble reactive phosphate (SRP) (for the control, both the TAN and SRP values were around 0 μM throughout the rearing), and (C) chromophoric dissolved organic matter (CDOM) concentrations using the *A*_λ325_ proxy, measured during the 10 days of rearing. Each bar of the bar plot corresponds to the average concentration of the triplicate tanks according to condition (with or without antibiotic) and to its standard deviation for each day of rearing. Time series of (D) larval stage with the theorical Larval Stage Index (LSI) to be reached each day and (E) larval survival during the experiment compared to the reference in turquoise (e.g., usual survival and stage obtained for a specific day; both the survival and stage reference werecalculated for each day using data from 10 years of successful rearing Ifremer data, Pham, personal communication.). Left side of panel D, larval stages indices of 0, 1, 2, 3, 4, 5, 6, and 7 correspond to nauplii, zoea stages 1 to 3, mysis stages 1 to 3, and post-larva stage, respectively. Control indicates the control water without larvae and food; With-1, With-2, and With-3 correspond to the rearing water with antibiotic in tanks 1, 2, and 3; and Without-1, Without-2, and Without-3 correspond to the rearing water without antibiotic in tanks 1, 2, and 3. D0 to D9 indicate the days of rearing.

Throughout the 9 rearing days, the temperature and salinity of all tanks remained constant at 30° ± 1°C and 35 ± 0.2 ppt, respectively. Total ammonia nitrogen (TAN), soluble reactive phosphate (SRP), and the absorption coefficient at 325 nm (*A*λ_325_, proxy for CDOM [chromophoric dissolved organic matter]) remained low and constant in the control waters whereas they increased constantly in the larval rearing waters, with or without antibiotic treatment (Fig. 1). Indeed, in the rearing water, TAN concentrations increased around 7-fold between day 3 (D3) and D9 in both treatments (with and without antibiotic) (Fig. 1A). The same trend was observed for SRP, with the same magnitude (Fig. 1B). The CDOM tripled from D0 to D9 in all larval rearing water tanks (Fig. 1C). Compared to the reference, significant mortality was observed between D6 and D9 regardless of the treatment. Overall, the two treatments resulted in survival rates below 45% on D9, except for the third tank with antibiotic (samples with D9-3), with a survival rate of 57% (Fig. 1D). At the end of the experiment, a delay in larval growth was observed because all larvae in all tanks were still in the mysis stage (Fig. 1E) compared to the reference, where at least half of the shrimp cohort usually reached the post-larvae stage at D9 (the stage reference is an average of the daily larval stage calculated using 10 years of data for successful rearing; Pham, personal communication). Mann-Whitney tests showed that the addition of antibiotic had no significant effect on TAN, SRP or CDOM concentrations, with *P* values of 0.851, 0.860, and 0.649, respectively. Furthermore, the addition of antibiotic did not impact larval development or larval survival, with *P* values of 0.276 and 0.786, respectively.

### Dynamic of the active microbial diversity in the rearing water.

We used a metabarcoding approach to characterize the prokaryotic communities associated with the water samples, resulting in, after filtration, chimera, chloroplast and mitochondrial removal, and taxonomic assignment, a total of 4,612,207 reads clustered into 1,563 OTUs according to the database Silva 132-16S. The OTUs were spread into 21 phyla, 3 from Archaea and 18 from Bacteria. The alpha diversity index values, shown in Table S1, indicated that the richness estimated using ACE and Chao1 was generally higher in the rearing water samples without antibiotic than in the rearing water with antibiotic and the control water. The richness was higher in the reservoirs than in the control water. The same trend was observed for the evenness estimated with the Shannon and Inverse Simpson indexes. The Mann-Whitney tests performed on the alpha-diversity indices between water samples on D1 with and without antibiotic indicated that diversity was not significantly different; whereas considering the entire set of samples (from D1 to D9) with and without antibiotic, the alpha diversity indices were significantly different, with *P* < 0.05.

**FIG 2:**
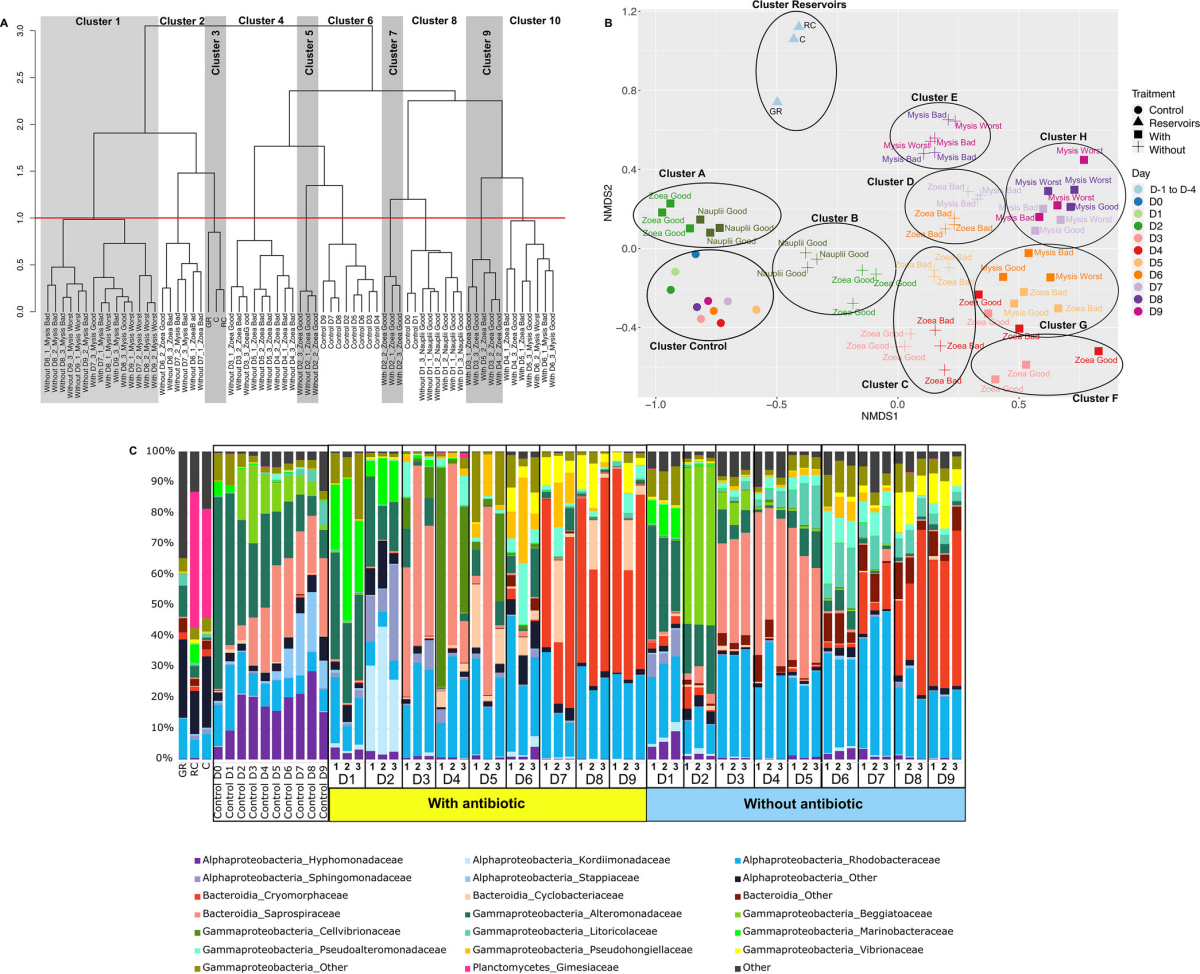
Clustering and microbial composition of the water samples with bacterial distribution at the family level in all the water samples. (A) Hierarchical clustering diagram based on microbial community dissimilarity, made at the operational taxonomic unit (OTU) level using the Bray-Curtis matrix. To define clusters, a threshold of 1 was set (red dotted line). (B) Nonmetric multidimensional scaling (NMDS), with stress at 0.15, allows visual representation of the rearing water samples according to the treatment (antibiotic or not), sampling day, and the larval stage and survival rate as labelled; clusters are represented by black circles. (C) Relative abundance of the main prokaryotic families. Relative abundance is shown as a percentage of the total prokaryotic sequences per sample. Only families representing more than 1% of the overall abundance in at least 3 samples are shown. GR, primary reservoir sample; RC, secondary reservoir sample; C, water circulated over 3 days through the filters and skimmer of the secondary reservoir; Control, control water without larvae or food. D indicates the sampling day, 1, 2, or 3, corresponding to the replicate tanks for the rearing water with or without antibiotic. Good survival: larval survival rate above, equal to, or slightly below the reference (less than 5%). Bad survival rate: survival rate between 50% and 100% of the reference value. Worst survival rate: survival rate below 50% of the reference value.

We first visualized how the samples clustered together according to the entire normalized OTU table. The dendrogram displayed 10 clusters at a threshold of 1, with clear distinction among the samples regarding the water type: control, rearing water with antibiotic, rearing water without antibiotic, and reservoir water. Clusters 1 and 8 were the exception, as they included samples with and without antibiotic (Fig. 2A). Indeed, cluster 1 encompassed all the rearing water samples without antibiotic collected on D8 and D9 and all the rearing water with antibiotic samples between D7 and D9. Cluster 2 pooled the water samples without antibiotic collected on D6 and D7. The water reservoirs (primary [GR] and secondary [RC and C]) were gathered together in cluster 3. Clusters 4, 5, and 6 encompassed all the rearing waters without antibiotic sampled from D2 to D9; while clusters 7, 9 and 10 gathered all the rearing waters with antibiotic sampled from D2 to D9. Cluster 8 was composed of all the rearing water samples collected on D1. As pictured in Fig. 2A, the clustering was mostly consistent with the larval stages, while survival status did not influence grouping.

Nonmetric multidimensional scaling (NMDS) displayed a clear distinction between the water reservoirs (cluster reservoirs), the control water (cluster control), and the rearing water samples with (clusters A, F, G, and H) or without antibiotic (clusters B, C, D, and E) as shown in Fig. 2B. However, the NMDS did not gather the samples as in the dendrogram; for example, cluster A encompassed the rearing water with antibiotic collected on D1 and D2, while in the dendrogram these samples were grouped in cluster 8 with the control samples collected on D0 and D1 and the rearing water without antibiotic samples collected on D1 and D2. However, like the dendrogram, it showed a daily evolution according to the sampling time and no clear grouping according to larval survival rate.

The active microbiota of the water samples showed different patterns according to the presence or absence of larvae or antibiotic (Fig. 2C). The primary reservoir (GR) which contained the lagoon seawater was mostly composed of members of *Alphaproteobacteria*, *Gammaproteobacteria*, and *Bacteroidota* classes and lineages related to the *Rhodobacteraceae* and *Alteromonadaceae* families. The secondary reservoirs (RC and C), before (RC) and after (C) recirculation through filters, exhibited the same outlines, with a high abundance of the *Gimesiaceae* family, which comprised around or less than 1% of the relative abundance in the rearing water sample and the control (Fig. 2C). The control water without larvae, antibiotic, or food was mainly composed of *Hyphomonadaceae*, *Alteromonadaceae*, and *Saprospiraceae*, with their relative abundances varying over the 10 days. The microbial composition of the rearing water with and without antibiotic displayed a homogeneous distribution among the replicates throughout the whole rearing, as shown in Fig. 2C. They were both mostly composed of *Rhodobacteraceae*, *Alteromonadaceae*, *Saprospiraceae*, *Litoricolaceae*, and *Cryomorphaceae* (Fig. 2C). On D2, and only on D2, the *Beggiatoaceae* were highly present in the water without antibiotic and the *Kordiimonadaceae* were found in the rearing water with antibiotic.

### Specific and core microbiomes of the rearing water.

**FIG 3:**
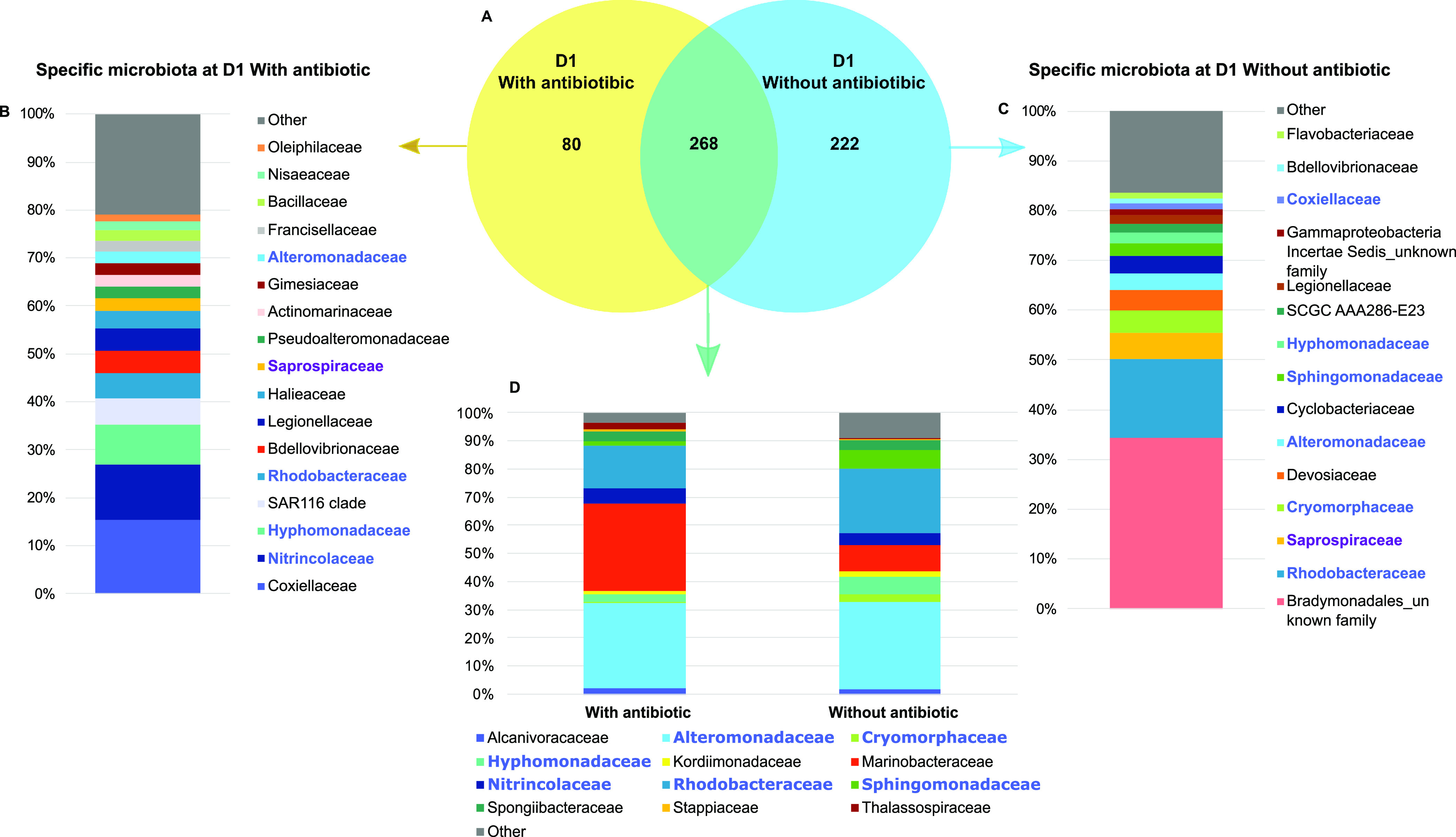
Microbial communities associated with specific and core microbiomes on D1 in the rearing waters supplemented or not supplemented with antibiotic. (A) Venn diagram of shared OTUs between the rearing waters with and without the antibiotic erythromycin. Blue ellipse represents OTUs common to the rearing water from the tanks without antibiotic collected on D1; yellow ellipse represents OTUs common to the rearing waters from the tanks with antibiotic on D1. The overlapping area between the blue and yellow ellipses represents the OTUs common to all water samples collected on D1. The numbers inside the ellipses and in the overlapping zones correspond to the total number of OTUs present under each condition. (B) Top 17 families with a relative abundance higher than 1% corresponding to the specific microbiome of the rearing water with antibiotic. (C) Top 15 families (when possible, higher level otherwise) with a relative abundance higher than 1% corresponding to the specific microbiome of the rearing water without antibiotic. (D) Top 12 families with a relative abundance higher than 1% corresponding to the core microbiome common to the rearing water with and without antibiotic collected at D1. Shown in blue are the families present in the core microbiome on D1 and in the specific microbiomes of the rearing water collected on D1 with and without antibiotic.

To determine the impact of the antibiotic erythromycin on the microbiome of the rearing water, we made a Venn diagram of the rearing waters on D1 with and without antibiotic (Fig. 3A). On D1, the rearing water hosted the nauplii which had a 100% survival rate, and these originated from the same nauplius batch; thus, we mostly expected to highlight the effect of the antibiotic on the rearing water microbiota. The water with antibiotic had 80 specific OTUs. These were mostly composed of members of *Coxiellaceae*, *Nitrincolaceae*, *Rhodobacteraceae*, and *Hyphomonadaceae* families and the SAR 116 clade (Fig. 3B). A total of 222 OTUs were specific to the water without antibiotic on D1, accounting for an average of 4% of the relative abundance, and these were dominated by an unknown family of the *Bradymonadales* and by *Rhodobacteraceae* (Fig. 3C). A core microbiome of 268 OTUs was shared in the rearing waters without or with antibiotic. This core microbiome encompassed an average of 97% of the reads. These 268 OTUs were mostly related to the *Marinobacteraceae*, *Alteromonadaceae*, and *Rhobodacteraceae* (Fig. 3D).

**FIG 4:**
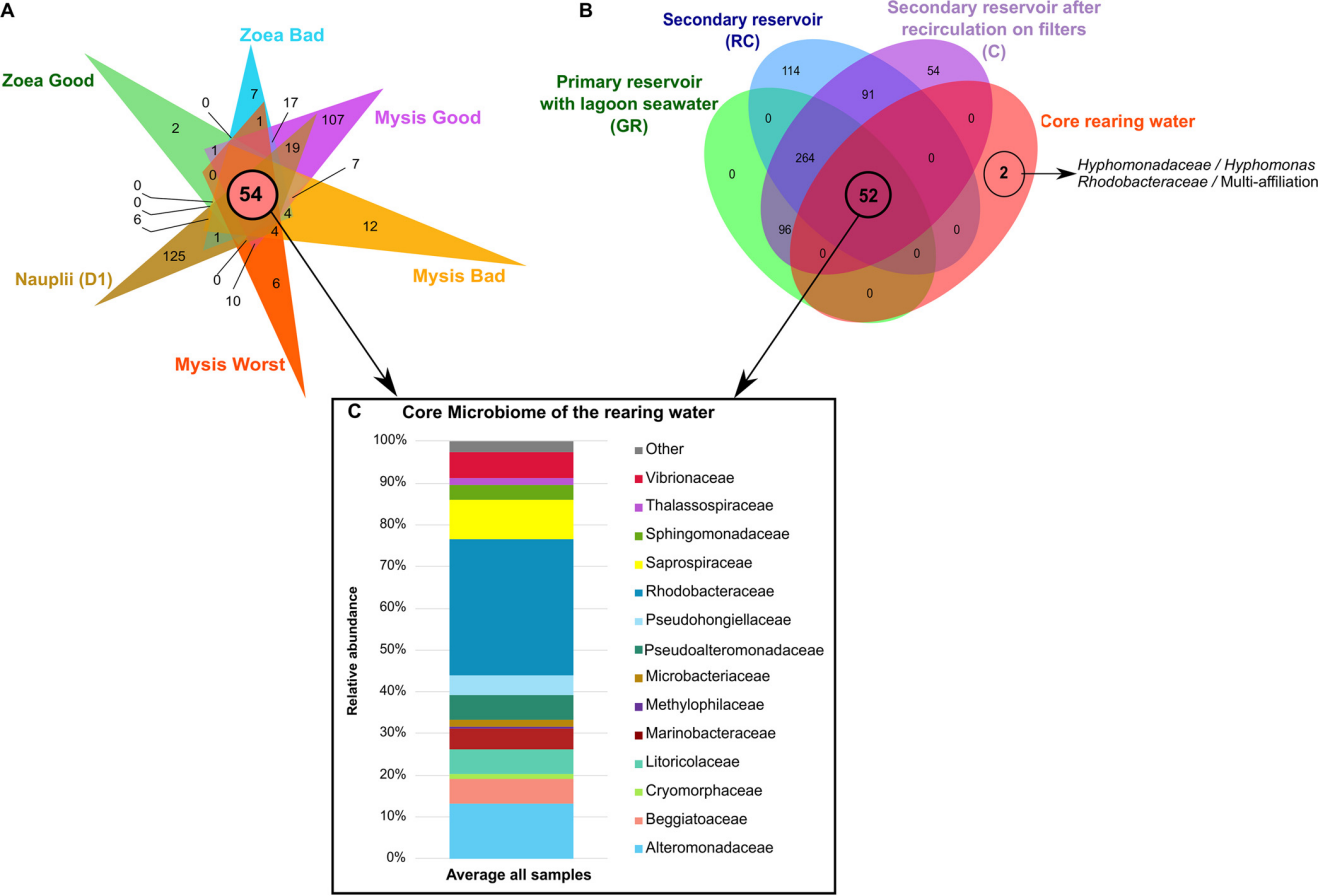
Microbial communities associated with the specific and core microbiomes of the entire rearing experiment in the rearing water. (A) Venn diagram of shared OTUs among rearing water samples. The overlapping area between all the ellipses, represented by a salmon circle, corresponds to the core microbiome composed of the 54 OTUs common to all samples. The numbers inside the ellipses and in the overlapping zones correspond to the total number of OTUs present under each condition. (B) Venn diagram of shared OTUs among the core rearing water samples and water storages. The numbers inside the ellipses and in the overlapping zone correspond to the total number of OTUs present under each condition. (C) Histogram of the families with relative abundance higher than 1%, constitutive of the core microbiome of all samples. All families with a relative abundance lower than 1% are included as “Other.”. Barplot was constructed by averaging all rearing water samples.

As described previously, the water samples were grouped according to larval stage and sampling day regardless of health status (Fig. 2A and B). However, we wondered whether several OTUs were specific to a given larval stage and health status and whether all the rearing water samples shared a core microbiome. Thus, in addition to the core microbiome on D1, several core microbiomes specific to a given condition were compared (Fig. 4): water hosting the zoea with a good survival rate (Zoea Good), water with the zoea with a bad survival rate (Zoea Bad), water with the mysis with a good survival rate (Mysis Good), water with the mysis with a bad survival rate (Mysis Bad), and rearing water with the mysis with a low survival rate (Mysis Worst). A core microbiome of 54 OTUs was co-owned by all samples and was mostly composed of members of *Rhodobacteraceae*, *Alteromonadaceae*, *Saprospiraceae*, *Litoricolaceae*, *Beggiatoaceae*, *Pseudoalteromonadaceae*, and *Vibrionaceae* (Fig. 4A and C). This core microbiome was compared to the active microbiota of the water reservoirs, and the Venn diagram shows that 52 out of 54 OTUs of the core rearing water microbiome were also detected in the water storages. This indicated that both the lagoon (e.g., water from the primary reservoir) and storage water had significant roles during the rearing process. Our second observation was that only 2 OTUs, related to the *Hyphomonadaceae* and *Rhodobacteraceae*, were the signature of the rearing water. As we emphasized when we compared the core microbiome of the rearing water with that of the water reservoirs, several OTUs were co-owned between all water samples. As a consequence, we examined whether certain constitutive OTUs from the specific rearing water microbiome of a given condition could also be detected in the water reservoirs.

**FIG 5:**
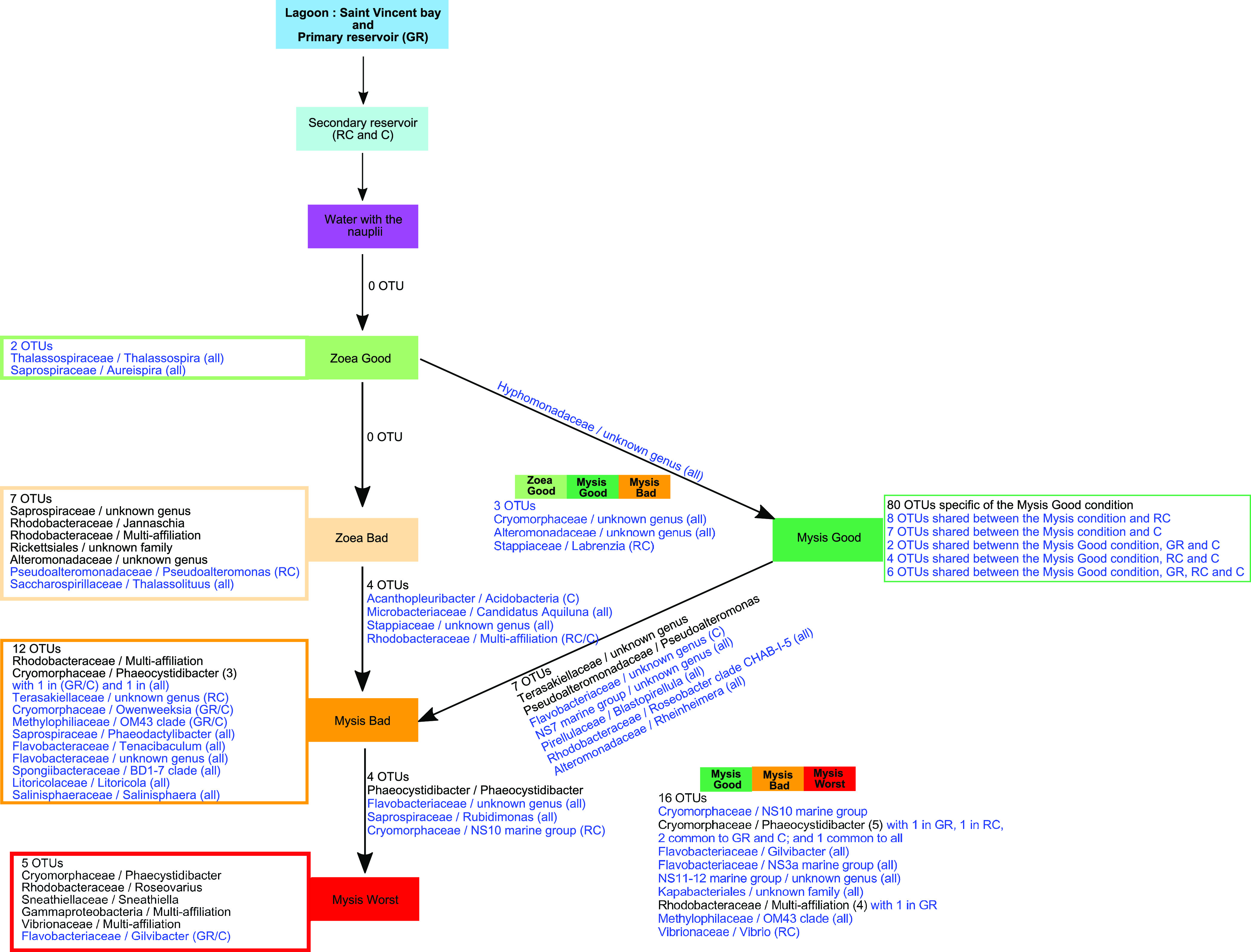
Microbial communities specific or common to one or several conditions, from the lagoon to the rearing water, throughout the rearing experiment. Schematic representation of the OTUs specific to a given condition or shared between 2 or more successive conditions (e.g., water hosting the Zoea Good, Mysis Good, and Mysis Bad conditions) from the storage to the rearing water corresponding to each larval stage. Lineages in blue were detected in one, two, or all storage waters; lineages in bold were detected in the primary reservoir (GR) that stored the lagoon seawater. The number of OTUs related to the same genus are in brackets after each OTU affiliation. Reservoir types: GR, primary reservoir containing the lagoon seawater; RC, secondary reservoir; and C, water which was circulated over 3 days through the filters and skimmer of the secondary reservoir. All stand for the 3 reservoirs (e.g., OTUs found in the 3 reservoirs).

The comparison is shown in Fig. 5, where several OTUs detected during the rearing at various specific stages and health statuses were also detected in the water reservoirs (Fig. 5, OTU in blue), and the lagoon seawater. For example, the 2 OTUs specific to the water hosting the “Zoea Good” condition were already present in the water storage; while of the 5 OTUs specific to the “Mysis Worst” condition, only 1 was also found in the water reservoirs. These observations indicated that many OTUs detected as specific lineages for a given condition were present in the lagoon and the water reservoirs and were reactivated during rearing according to water quality, larval stage, and/or larval health status.

### Differential analyses of microbial communities.

An initial linear discriminant analysis (LDA) effect size (LEfSe) analysis was performed to investigate the families which were significantly more abundant in the water according to rearing day. On D1, 3 families were prevalent in the rearing water, *Hyphomonadaceae*, *Marinobacteraceae*, and *Alteromonadaceae*. On D2, only one family, *Thalassospiraceae*, was enriched. The following day (D3), one biomarker was highlighted with the *Saprospiraceae* family. On D4, the samples were dominated by *Rhodobacteraceae*, while no biomarker was found on D5. On D6, the samples were enriched in *Pseudoalteromonadaceae*. The following day (D7), two biomarkers, *Microbacteriaceae* and *Pseudohongiellaceae*, dominated the rearing water. On D8, *Methylophilaceae* and *Vibrionaceae* were enriched in the rearing water; while at the end of the larval rearing, on D9, the water samples were enriched in *Cryomorphaceae*.

A second LEfSe analysis was conducted to expose the families which were significantly more abundant in the rearing water according to larval stage and health status. When the nauplii with a good survival rate were in the rearing water, the most prevalent biomarkers in the samples were the same as those exhibited at D1 in the previous LEfSe: *Hyphomonadaceae*, *Marinobacteraceae*, and *Alteromonadaceae*. The rearing water hosting the zoea with a good survival rate had 3 enriched families: *Saprospiraceae*, *Sphingomonadaceae*, and *Thalassospiraceae*. Except for *Sphingomonadaceae*, all of these were detected in the first LEfSe, with the *Saprospiraceae* highlighted on D2 and the *Thalassospiraceae* on D3. The *Litoricolaceae* and *Rhodobacteraceae* were enriched in the rearing water hosting the zoea with a bad survival rate. *Rhodobacteraceae* was also the prevalent family on D4. The biomarker specific to the mysis with a good survival rate in the rearing water was *Pseudohongiellaceae*, previously detected as prevalent biomarker on D7. When the mysis had a bad survival rate, the samples were dominated by the *Cryomorphaceae* and *Microbacteriacace*, families which were previously detected as biomarkers on D9 and D7, respectively. Three families were significantly enriched in the rearing water when it hosted the mysis with the worst survival rate, *Methylophilaceae*, *Pseuodoalteromonadaceae*, and *Vibrionaceae*, previously detected in the first LEfSe on D8 for *Methylophilaceae* and D6 for *Vibrionaceae* and *Pseudoalteromonadaceae*.

Next, we performed a Pearson correlation to determine which factor, hydrochemical parameters or larval stage and status, shaped the biomarkers highlighted with the LEfSe, the most abundant families in the rearing water. The correlogram displayed that the “Mysis Good” condition was not correlated with any family and that the *Cryomorphaceae* were correlated with all factors tested. The “Nauplii Good” condition was positively correlated with the *Marinobacteracea*, *Alteromonadaceae*, and *Hyphomonadaceae*; however, these families positively correlated with TAN and SRP concentrations, making it difficult to untangle the effect of the nauplii on the rearing water. However, because the *Marinobacteracea* exhibited a strong coefficient correlation with the nauplii, we suggest that they are linked to the presence of the nauplii in the rearing water. The “Zoea Good” factor was strongly positively correlated with the *Saprospiraceae* and *Thalassospiraceae*, slightly positively correlated with the *Alteromonadaceae*, and negatively correlated with the *Litoricolaceae* and *Vibrionaceae*. Because the *Thalassospiraceae* were slightly positively correlated with TAN and SRP and the *Saprospiraceae* were negatively correlated with SRP, we inferred that both the *Thalassospiraceae* and *Saprospiraceae* were linked to the “Zoea Good” condition. The “Zoea Bad” condition was positively correlated with the *Rhodobacteraceae* and *Litoricolaceae*, families that either did not correlate or slightly correlated with other factors. The “Mysis Bad” condition was slightly positively correlated with the *Microbacteraceae* and the *Vibrionaceae*. The *Microbacteraceae* were also moderately correlated positively with the CDOM proxy, while the *Vibrionaceae* were greatly correlated positively with both the “Mysis Worst” condition and the CDOM proxy. In addition, the “Mysis Worst” condition had a significant positive correlation with the *Methylophiliaceae*, which were also positively correlated with the CDOM proxy. Regarding the correlation coefficient, the *Methylophilaceae* appeared to be linked to the “Mysis Worst” condition. The *Alteromonadaceae* appeared to be positively correlated with increasing TAN and SRP, while the *Vibrionaceae* were positively correlated with increased CDOM (*A*λ_325_ proxy).

## DISCUSSION

To explore whether the microbiome of the rearing water was linked to the survival rate of P. stylirostris larvae, we tracked the active microbial diversity of the water storage and rearing water of 7 tanks during larval development: 3 with larvae treated with antibiotic, 3 with larvae not treated with antibiotic, and 1 with only water. The meta-analysis aimed to investigate whether specific OTUs were key lineages and whether families could be used as biomarkers of good or bad survival rates. Hydrochemical analyses were also performed to monitor the behavior of the dissolved organic matter, dissolved total nitrogen, and reactive phosphorus throughout the larval rearing.

In shrimp hatcheries worldwide, antibiotics are regularly used for prophylactic purposes (under veterinary advice) or to prevent larval mortality due to pathogenic *Vibrio* species (26, 27). At the Station Aquacole de Saint Vincent shrimp hatchery, erythromycin was added into the rearing water of 3 tanks on day 0 (before nauplii addition) and days 3, 5, 7 and 9 to determine whether it could increase larval survival and larval stage metamorphosis. In this experiment, we evaluated that neither larval development nor larval survival rates were dependent on the addition of antibiotic, likewise for the alpha diversity indices of the microbiota of the rearing water on D1 with the nauplii. This is interesting because erythromycin is known to target a large spectrum of bacteria, inhibit bacterial growth, and exhibit bacteriostatic effect (28). In our case, at least in the water on D1, only the rare biosphere was affected, as less than 3% of the active microbiome was specific to the condition with or without antibiotic. At the time of writing, only one study has examined the effect of antibiotic on the microbiome of the shrimp *Macrobrachium rosenbergii* at the zoea stage (29). The authors showed that the diversity indices did not show any significant differences between the water control and the water in which zoea had been reared during 3 days in the presence of oxytetracycline; this was similar to what we found in our samples on D1. Studies on erythromycin resistance in both bacterial strains and biofilms have shown that several gammaproteobacterial strains are less sensible to erythromycin than other taxa related to the *Actinobacteria*, *Alphaproteobacteria*, or Bacteroidetes (30). This may explain why taxa affiliated with the *Gammaproteobacteria* were more abundant in the rearing water with antibiotic on D1 (Fig. 2 and 3). However, this changed when we considered the whole rearing microbiome because alpha diversity indices were significantly different between the samples with or without antibiotic. This was consistent with the dendrogram and NMDS (Fig. 2). Indeed, both the dendrogram and NMDS grouped all the rearing water samples with antibiotic together into several clusters (with a threshold of 0.5 for cluster 1 in the dendrogram). However, as larval mortalities began to really occur at the Zoea stage on D3 to D4 in all tanks with or without antibiotic, it became difficult to untangle the effect of antibiotic from that of larval mortality on the rearing water microbiome.

**FIG 6:**
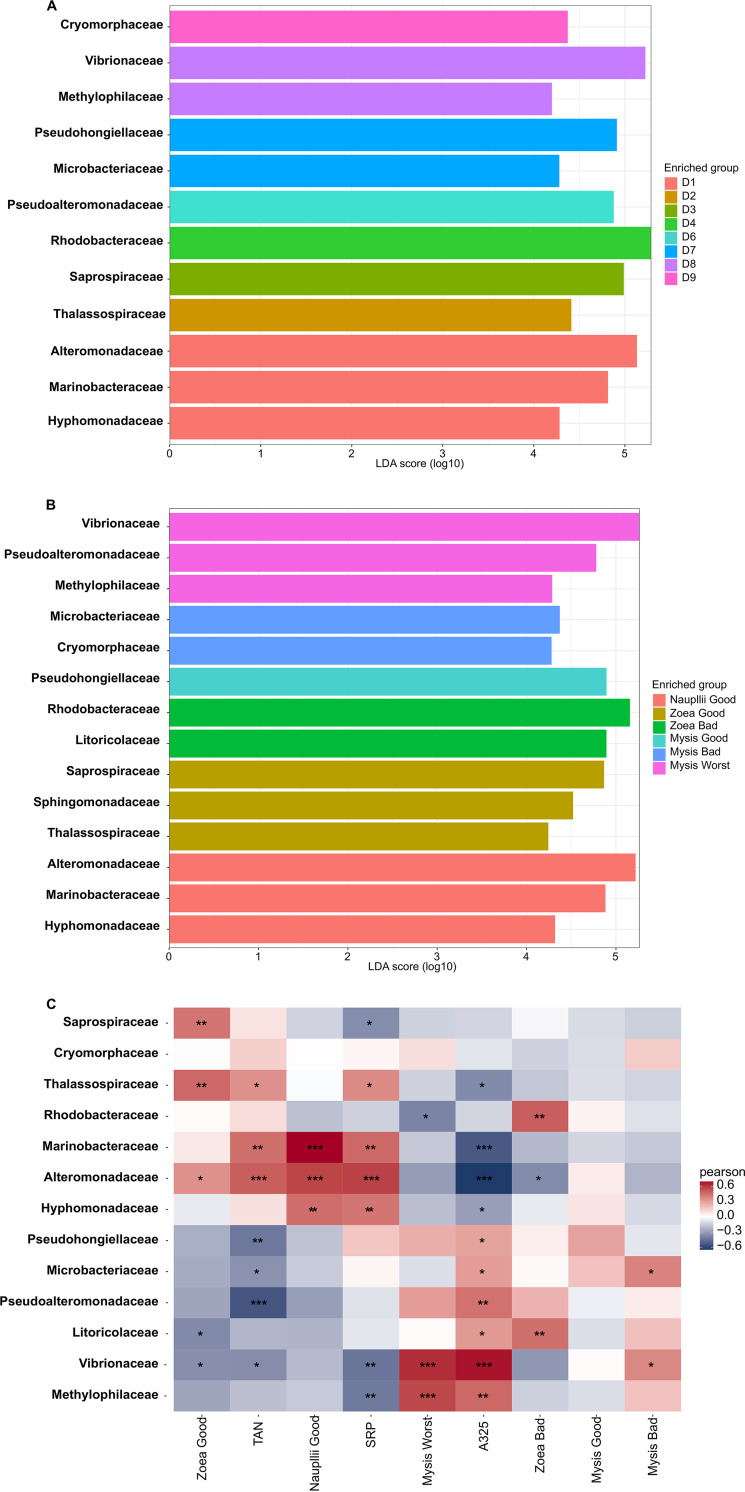
Differentially abundant families according to the rearing day, to the larval growth and survival status; and Pearson correlations between the biomarkers, hydro-chemical parameters of the rearing water and larval growth and survival status. (A) LEfSe (linear discriminant analysis [LDA] effect size) showing the families which were significantly more abundant in the rearing water according to rearing day from days 1 to 9. (B) Family-level biomarkers significantly more abundant in the rearing water according to larval growth and survival status. (C) Correlogram comparing biomarkers at the family level highlighted by LEfSe, with the following hydrochemical parameters: TAN, SRP, and *A*_325_ (stands for *A*_λ325_) proxy of the CDOM. Larval growth and survival status: Nauplii Good, Zoea Bad, Zoea Good, Mysis Good, Mysis Bad, and Mysis Worst.

Throughout the larval rearing, a global eutrophication of the water was observed compared to that in the control water which remained in the oligotrophic condition. Here, eutrophication was evaluated in the dissolved fraction with CDOM, TAN, and SRP measurements and was mostly due to particles from the larval leftover feed, feces, and exoskeleton as well as dead larvae and absence of water renewal. The CDOM, TAN, and SRP concentrations in the larval tanks were irrespective of antibiotic presence, larval stage, or larval survival rates (Fig. 1). The active microbial diversity dynamic over the rearing period seemed to be linked to the gradual water eutrophication and the larval stages as exhibited by the dendrogram, NMDS (Fig. 2A), LEfSe, and correlogram (Fig. 6A and C). This is linked to an overall increase in the proportion of *Bacteroidia* accompanied by decreases in *Alpha*- and *Gammaproteobacteria* (Fig. 2C); these classes were also predominant in the rearing water of P. vannamei (8) and P. monodon breeding (9). At the beginning of the experiment, the predominant families were *Alteromonadaceae* and *Marinobacteraceae*, and at the end, it was *Cryomorphaceae*. The fact that the *Alteromonadaceae* were highly represented in the water at the start of the rearing, positively correlated with TAN and SRP concentrations and negatively correlated with the CDOM proxy (Fig. 6C), and were even present in the control water is quite unusual because they are generally found in nutrient-sufficient to nutrient-rich environments (31, 32). This family has been found in one other shrimp larval rearing water, but at lower proportions than in our study (8). In this study, the *Alteromonadaceae* could play a significant role in larval health and/or in carbon biogeochemical cycle in the rearing water by metabolizing and removing some of the labile or semi-labile dissolved organic compounds and by breaking down organic matter molecules (33–35). Members of the family *Cryomorphaceae* are often detected in organic-rich oceanic water (36), so it was not surprising that they encompassed the most lineages at the end of the experiment in the eutrophic water (Fig. 2C and 6A). The relative abundance of the *Rhodobacteraceae* was highly distributed (>10% in most of the samples) during the entire rearing period; this was similar, but at a lower magnitude, to the findings of Zheng et al. 2017 (8). Members of the *Rhodobacteraceae* were found throughout all steps of the rearing process, from the storage water to all samples and even in the control, which was not surprising because *Rhodobacteraceae* has been defined as a keystone lineage in aquatic ecosystems (37). That fits with the suggested significant role of the *Rhodobacteraceae* as keystone taxa in the rearing water proposed by Zheng et al. (8). We have also shown that through the entire rearing, several taxa present in the primary reservoir, so in the lagoon, and in the secondary reservoirs, were not active in some intermediate sampling points and were found back active later in the rearing water highly suggesting a role of the lagoon water composition during the rearing. These data also suggested the activation of certain lineage according to the rearing condition such as the eutrophication of the water, larval development and/or survival (Fig. 5). Then, it appeared that the larval development and health also seemed to drive the dynamic of the active microbiome. In order to distinguish the effect of the rearing conditions hydro-chemical parameters or larval stage and health, on the main families highlighted with the LEfSe, we performed Pearson correlations (Fig. 6). The correlogram shows that the *Alteromonadaceae* and *Vibrionaceae*, respectively, were linked to increased ammonia, nitrogen, and soluble reactive phosphate or to increased colored dissolved organic matter in the rearing water. It also shows that the *Rhodobacteraceae*, for example, were positively correlated with the “Zoea Bad” condition. This contrasts with the keystone role of *Rhodobacteraceae* in the rearing water as described above. However, this correlation was made from both the core microbiome and the LEfSe data, meaning that when we only examined the shared microbiota among the samples, the *Rhodobacteraceae* were statistically enriched in the “Zoea Bad” condition. In addition, one could not deny that, for the entire rearing, the *Rhodobacteraceae* were always present regardless of larval stage or health status, highlighting their significant role.

**FIG 7:**
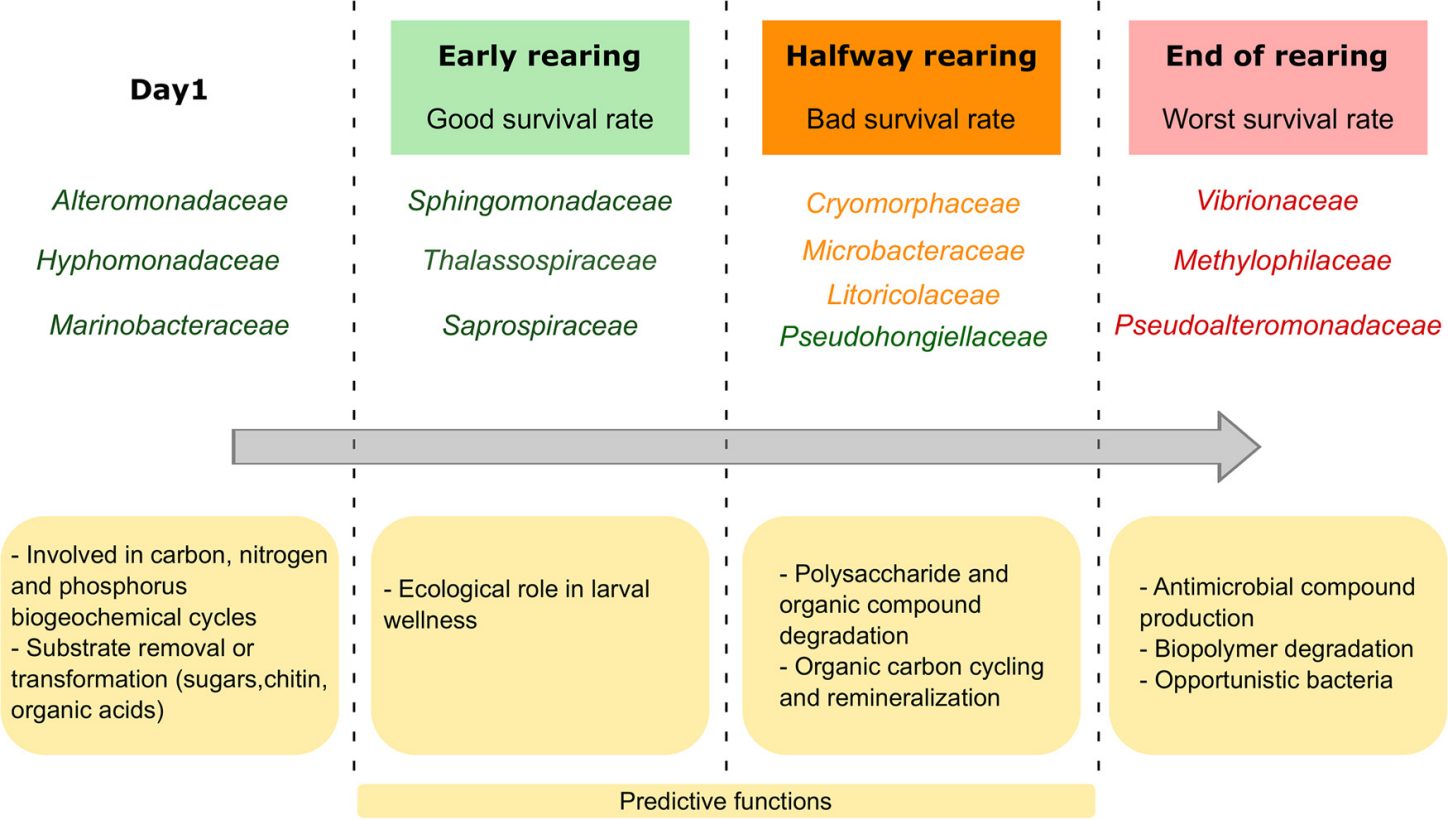
Schematic representation of the evolution of the microbial communities and their predictive functions, according to larval survival rate during rearing. Green: families detected in the rearing water when the larvae had a good survival rate. Orange: families detected in the rearing water when the larvae had a bad survival rate. Red: families detected in the rearing water when the larvae had the worst survival rate.

Our hypothesis to understand the mass larval mortality was that a shift in the active microbial diversity of the rearing water could hamper the larvae and lead to high larval mortality. We therefore explored whether biomarkers at the family level were associated with a certain mortality rate at a given larval stage and whether these biomarkers could ultimately be used to monitor larval health. The biomarker investigation revealed the prevalence of the *Hyphomonadaceae*, *Marinobacteraceae* and *Alteromonadaceae* in the “Nauplii Good” condition (Fig. 6B). These 3 families could be involved in the carbon, nitrogen, and phosphorus biogeochemical cycles by removing or transforming various substrates such as sugars, chitin, and organic acid (33–35, 38, 39) during the early stage of rearing. However, the Pearson correlation showed that only the *Marinobacteraceae* were a biomarker related to the “Nauplii Good” condition (Fig. 6C). Two families were found as biomarkers of the “Zoea Good” condition, the *Thalassospiraceae* and the *Saprospiraceae* (Fig. 6B), as also confirmed by the Pearson correlation (Fig. 6C). They seemed to have an important ecological role in larval wellness. Indeed, members of the *Thalassospiraceae* are known to be able to degrade complex carbon sources (40) or hydrocarbon and xenobiotic molecules (41, 42). The “Zoea Bad” condition had 2 biomarkers, the *Litoricolaceae* and the *Rhodobacteraceae*, as shown by the LEfSe and correlogram (Fig. 6B and C). Few data are available on *Litoricolaceae* except that they grow on oligotrophic medium; this could explain why they were prevalent during this stage of rearing (43). Interestingly, it has been shown that marine *Rhodobacteraceae* can produce antibacterial compounds or secondary metabolites that influence both the producing strain and the surrounding microbiota (44). Thus, the *Rhodobacteraceae* might contribute to the beginning of dysbiosis, as the larvae started to die under this condition; by producing antimicrobial compounds, they act on the rearing water microbiota to overcome r-strategist microorganisms and/or putative pathogens. The LEfSe displayed that the *Pseudohongiellaceae* were the biomarker for the “Mysis Good” condition; while the correlogram showed no correlation between any families and the “Mysis Good” condition. So far, *Pseudohongiellaceae* have been detected in the gut of the silver carp fish (45), in marine sediments (46), and in seawater (47). According to the genome annotations of several *Pseudohongiellaceae* species, they can degrade bio-polymers (48). This could explain their enrichment at that point of the rearing, where the mortality rate was still low and did not affect the main families. The biomarkers related to the “Mysis Bad” condition were the *Cryomorphaceae* and the *Microbacteraceae* (Fig. 6B). However, the Pearson correlation showed that only the *Microbacteraceae* were positively correlated with the “Mysis Bad” condition (Fig. 6C). This is surprising because in a previous study by Zheng et al. (8), the *Microbacteraceae* were the main contributors during the post-larval stage of rearing. The “Mysis Worst” condition included 3 biomarkers, *Methylophilaceae*, *Pseudoalteromonadaceae*, and *Vibrionaceae*, with the LEfSe (Fig. 7B), while the Pearson correlation showed a strong positive correlation with the *Methylophilaceae* and the *Vibrionaceae*; the latter was also highly positively correlated with the chromophoric dissolved organic matter (Fig. 6C, *A*_325_). The biomarker *Pseudolateromonadaceae* was also positively correlated with the chromophoric dissolved organic matter (Fig. 6C). The *Methylophilaceae* are methylotrophs (49). They have been detected in the gut of the sea cucumber *Apostichopus japonicus*, where it has been suggested that they help degrade organic matter, reduce the toxic effects of certain intestinal contents, and maintain gut health by influencing the intestinal microbiota (50). In our study, the *Methylophilaceae* might also act to reduce the amount of organic matter in the rearing water, as they were also positively correlated with the CDOM proxy; however, this biomarker exhibited the best correlation with the “Mysis Worst” condition. The *Vibrionaceae* and *Methylophilaceae* were positively correlated with the “Mysis Worst” condition and the CDOM proxy (Fig. 6C). Vibrios are often associated with shrimp rearing, either in the rearing water and/or the shrimp microbiome at all life stages, from eggs and larvae to juveniles, adults and broodstock, and in both healthy and diseased shrimps (8, 9, 11, 17, 51). Several *Vibrio* species are known to be shrimp pathogens in shrimp culture (52, 53). In New Caledonia, 2 vibrios are known: Vibrio nigripulchritudo (5) and *V. penaecida* (54). They cause juvenile and adult mass mortality but do not affect larvae (55). In our study, members of the *Vibrionaceae* family were present in all samples from the lagoon/primary reservoir to the control and the rearing water supplemented or not with antibiotic (Fig. 2C). Because many *Vibrio* species are opportunistic bacteria with an r-strategy (56), they might have outcompeted the other communities that were hampered by the unbalanced microbiome of the rearing water due to the larval mortality since the zoea stage. The *Pseudoalteromonadaceae* were statistically highly enriched in the rearing water on D4 (Fig. 6A) and in the rearing water of the mysis with the worst survival rates (Fig. 6B). These, then, may contribute to hamper dysbiosis of the water microbiota, because the mortality started on D3, overcoming r-strategist microorganisms and/or putative pathogens by producing antimicrobial compounds. Indeed, they might have been released in the rearing water from the dead larvae as the number of dead larvae in the water increased. On the other hand, the *Pseudoalteromonadaceae* are found in various marine environments and many species produce molecules with bactericidal effects (24, 57). Previous studies have shown that taxa affiliated with the *Pseudoalteromonadaceae* are present in the gut of both healthy and diseased shrimps (58) and in their rearing environment (59). Other studies have shown that *Pseudoalteromonadaceae* are opportunistic bacteria, and they may have overpassed the core microbiota of the rearing water by the end of the rearing (60, 61). Like the *Vibrionaceae*, the *Pseudoalteromonadaceae* and the *Methylophilaceae* were present from the lagoon/primary reservoir throughout the whole rearing process (Fig. 2C), indicating that they found suitable growth conditions, like many other taxa that were present in the reservoirs and then activated again later during the rearing process. Altogether, these biomarker data allowed us to sum up the time series of the evolution of the biomarkers during the rearing process, as shown in Fig. 7.

Taken together, our results indicate that the active microbiota of the rearing water is highly dynamic throughout the rearing process, with distinct communities influenced by the progressive eutrophication of the water, larval stage, and larval survival rate. Microbial families related to water eutrophication were highlighted. The *Alteromonadaceae* were related to increased ammonia, nitrogen, and soluble reactive phosphate, while the *Vibrionaceae* and *Methylophilaceae* were linked to increased colored dissolved organic matter in the rearing water. Many OTUs were shared between the primary reservoir containing lagoon seawater, the secondary reservoirs (both RC and C samples), and the core microbiomes of the rearing waters. This provides evidence of the activation of certain lineages according to water composition and/or larval stage and health and is also evidence of the influence of the lagoon water initial composition on the larval rearing water microbiome. Biomarkers at the family level were revealed according to a given larval stage, larval health status, and specific microbiome (Fig. 7). This suggests that each larval stage and status condition has its specific microbial signature in the rearing water. In addition, our findings provide evidence of biomarkers which could be used as sentinels to monitor larval health during the early stages of rearing. Indeed, *Marinobacteraceae* were specific to the “Nauplii Good” condition, *Thalassospiraceae* and *Saprospiraceae* to the “Zoea Good,” *Litoricolaceae* to the “Zoea Bad,” *Microbacteraceae* to the “Mysis Bad,” and *Methylophilaceae* to the “Mysis Worst.” We eliminated the *Vibrionaceae* as biomarkers of the “Mysis Worst” condition because they had a stronger correlation with dissolved organic matter. The *Microbacteraceae* and *Methylophilaceae* could be potential announcers of upcoming and progressive larval mortality. Indeed, their prevalence and abundance in the rearing water, or even earlier in the lagoon or reservoir, might indicate the future fate of larval rearing. Thus, the highlighted biomarker families might be used as potential early warning sentinels in water storage to infer the evolution of the larval rearing during its early days. Potential future steps could be developed, such as designing PCR or qPCR primers specific to these key lineages to test the lagoon water before rearing and during the early days of rearing. Ultimately, we may eventually be able to manage the active microbiota of the rearing water, which is a major concern in aquaculture (62), and select beneficial microorganisms to improve larval shrimp rearing.

## MATERIALS AND METHODS

### Larval shrimp rearing.

The experiments were carried out in November 2018 in an experimental shrimp hatchery hosted in a shrimp farming research facility at the Station Aquacole de Saint Vincent (Boulouparis, New Caledonia). The reproduction of P. stylirostris was performed by artificially inseminating mature females as described by Pham et al. (63). The day after hatching, nauplii were transferred into 100-L rearing tanks at a density of 180 nauplii/L. The water salinity was 35 ppt, the temperature was 30°C, and a 14:10 h day:night photoperiod was applied. From the zoea 1 to zoea 2 stages, larvae were fed with microparticles six times per day and supplemented with frozen *Tetraselmis* sp. twice a day. For larvae from the zoea 3 stage to the post-larvae stage, in addition to microparticles, living *Artemia* sp. nauplii (from 20 to 40 nauplii/larvae/day) were administered twice per day instead of frozen *Tetraselmis* sp. Throughout the 10 days of the larval rearing, there was no water exchange. The seawater was collected from the Saint Vincent Bay (Boulouparis, New-Caledonia) to a primary reservoir through a pumping system with pores of 1 cm. Next, the water was filtered through a sand filter and a 10-μm filter prior to storage in a secondary reservoir, where it was continuously circulated through a skimmer and a series of 10- and 5-μm filters over 3 days. The day following the fecundations, the 100-L rearing tanks were filled with water which had passed through a 1-μm filter and been treated with UV. This water followed the same path as described by Giraud et al. (17). EDTA at 5 g·m^−3^ was added in all tanks. An increasing intensive bubbling was implemented in each tank to allow good oxygenation of the water. The experiment was performed in triplicate for each condition except for the control. Thus, 7 tanks were monitored during the experiment: 3 larval tanks with erythromycin added at 2 ppm a few hours after the EDTA (day 0) and on days 3, 5, 7 and 9; 3 tanks with larvae but without antibiotic; and 1 tank (control) without larvae, antibiotic, or food.

### Water sample collection and storage.

Prior to the start of the experiment, 1 L of lagoon seawater from the primary reservoir was collected for both chemical and microbial diversity analyses (GR sample). Next, just after being pumped, 1 L of water from the secondary reservoir was collected (RC sample) after an additional 3-day circulation through the skimmer and filters of the secondary reservoir (C sample). The tanks were filled the day after the fecundations and, after addition of EDTA, 1 L was collected in the control tank (sample control, D0). The nauplii were introduced to the tanks on D0 and sample collection began on D1. Throughout the 10 days of the experiment, 1 L of water was collected daily from each tank in the morning before the 1st feeding of the day (6 AM). Samples were collected in opaque containers and immediately kept on ice until filtration. The water samples were filtered through 0.22-μm sterile membrane filters (S-Pak, MilliporeSigma, Burlington, MA). In order to examine the active microbial diversity, the filters were directly stored at −80°C. The filtrates were distributed and stored accordingly: 50 mL was poured into a pre-combusted (8 h, 450°C) glass bottle for chromophoric dissolved organic matter (CDOM) analysis and kept in the dark until further analysis done within the hour after the sample collection; 40 mL for the total ammonium determination and 40 mL for the soluble reactive phosphate, both stored in acid-washed plastic vials and put at −20°C until analysis. A total of 67 samples were collected throughout the rearing in addition to the 3 storage water samples. The rearing and control waters were named as follows: “control” for samples collected from the control tank; “with” for samples collected from tanks with antibiotic and “without” for those collected from tanks without antibiotic, followed by the sampling day (D0 indicating the day after the fecundation, which corresponds to the day where the nauplii were placed in the rearing tanks; to D9, indicating the 10th day of rearing) and the replicate numbers 1 to 3 corresponding to the triplicates.

### Daily determination of the zootechnical parameters: shrimp larval stages and survival rates.

Every day, larval stages and survival rates were determined by examination under a binocular magnifying glass. The Larval Stage Index (LSI) was calculated as follows, based on the equation made by Maddox and Manzi (64) but modified to consider all life stages:

(1) $$\text{LSI }=\;\left(\left[0\times N_{\mathrm{ii}}]+[1\times \text{Z1}]+[2\times \text{Z2}]+[3\times \text{Z3}]+[4\times \text{M1}]+[5\times \text{M2}]+[6\times \text{M3}]+[7\times \text{PL}\right]\right)/N$$

where *N*_ii_ is the number of larvae observed during the nauplius stage, Z1 indicates the number observed during the zoea 1 stage, Z2 is zoea 2, Z3 is zoea 3, M1 is mysis 1, M2 is mysis 2, M3 is mysis 3, PL is post-larval 1, and *N* is the total number of observed larvae (minimum 30). The Larval Survival Rate (LSR) was determined by averaging 3 direct counts of the living and dead larvae in 1 L of rearing water per tank and per day. The LSR was determined as follows: LSR =100 × counted living larvae/initial number of nauplii.

### Hydrochemical analysis.

The temperature and salinity in each tank were monitored twice a day (morning and afternoon) using the probe pH/Cond 3320 (Mettler Toledo, Columbus, OH). The absorption spectra of the chromophoric dissolved organic matter was recorded directly at wavelengths from 200 to 700 nm at 1-nm intervals using a 10-cm quartz tank and a Shimadzu UV-1800 spectrometer (65). MilliQ water was used as the reference. The absorption coefficients at 325 nm (*A*_λ325_), which was a proxy for CDOM concentration, were used to describe the evolution of CDOM in the tanks (66). Concentrations of TAN and SRP in the filtered water were analyzed using the methods described by Holmes et al. (67) and Murphy and Riley, respectively (68).

### RNA extraction, reverse transcription, sequencing method, and sequence processing.

RNAs of the water samples were extracted from the filters using the RNAeasy Powerwater kit (Qiagen, Hilden, Germany) following the manufacturer’s instructions. Then, the RNAs were reverse-transcribed into cDNAs using 10 μL of RNA (200 ng/μL), 1 μL of the reverse transcriptase M-MLV at 200 u/μL (Promega, Madison, WI), 2 μL random hexamers at 50 μM, 4 μL of 5× buffer, 2 μL of a mix of deoxynucleoside triphosphate at 10 mM each, and 1 μL of RNase/DNase free water. The reverse transcriptions were carried out on a Veriti instrument (Applied Biosystems, Waltham, MA) using the following program: 10 min at 25°C, 2 h at 42°C, and 5 min at 85°C. The cDNAs were stored at −80°C until shipping to MrDNA (Molecular Research LP, Shallowater, TX), where PCR, barcode indexing, and sequencing of the V4 hypervariable region of the reverse-transcribed bacterial 16S rRNA molecule were conducted. PCRs were performed for each sample using the universal primer combination 515F and 806R (69, 70). The sequencing was performed using Illumina HiSeq technology with 2 × 300 bp chemistry following MrDNA protocols. The raw prokaryotic 16S rRNA gene sequencing data from HiSeq sequencing were processed using the pipline Find, Rapidly, OTU with the Galaxy Solution (FROGS) version 2.0.0 (71). In brief, all reads first were trimmed by length and assigned to a specific sample based on barcode recognition using the demultiplex tool in FROGS. Next, the sequences were clustered using the SWARM algorithm with an aggregation distance of 3. Chimeras were removed using the Vsearch algorithm. An additional filtering step for the abundance was used at a threshold of 0.005% (72) to select the most relevant OTU sequences. Taxonomic affiliation was assigned by BLASTn+ using the Silva database 132-16S (73).

Diversity analyses were performed after removing the chloroplast and the mitochondria sequences.

### Statistical analyses and Venn diagrams.

The alpha diversity, ACE, Chao 1, Shannon, and inverse Shannon indices were calculated for each sample using FROGSSTAT Phyloseq in Galaxy. Mann-Whitney tests were performed using XLSTAT (Addinsoft, Paris, France), a statistic tool appended to Microsoft Excel, on the alpha diversity indices to assess significant differences between the following treatments: rearing water with versus without antibiotic on D1, rearing water versus without antibiotic throughout the rearing, control versus rearing water with antibiotic, and control versus the rearing water without antibiotic. Mann-Whitney tests were also performed to determine the impact of the absence or presence of antibiotic on the chemical values (TAN, SRP, and CDOM) and on larval stages and survival rates. Results with a *P* value below 0.05 were considered statistically significant. Prior to microbiota analysis, the sequencing data were normalized with the count per million (CPM) method using the edgeR package in R Studio software. Beta diversity was visualized using an agglomerative hierarchical clustering method and an NMDS plot. The dendrogram was built using a Bray-Curtis dissimilarity matrix and the agglomeration method of Ward (74), using the Vegan package (75) in R Studio. The NMDS and Bray-Curtis distances were built using the packages phyloseq (76) and ggplot2 (77) in R Studio. For further statistical analyses, we divided the larval survival rates into 3 groups as follows: good survival rate corresponding to a larval survival rate (LSR) above, equal to, or slightly below the reference (less than 5%) (the reference is an average survival rate calculated for each day using data from 10 years of successful rearing; Ifremer data, Pham, personal communication); bad survival rate corresponding to a larval survival rate between 50% and 100% of the reference value (e.g., an LSR of 60% is a bad survival rate when the reference, on the same day, is at 80%); and worst LSR displaying a survival rate below 50% of the reference value. Several Venn diagrams (78) were constructed using the Jvenn interactive tool (79) to highlight specific microbial communities and particular patterns specific to given conditions. An initial Venn diagram was made to compare the impact of the antibiotic on the active microbial diversity in the rearing water on D1 with the nauplii before the start of larval mortality. From this diagram, we created a core microbiome of the rearing water hosting the nauplii with a good survival rate. Next, several core microbiomes were constructed specific to the following conditions: corresponding to the water hosting the zoea with a good survival rate (Zoea Good), corresponding to the water with the zoea with a bad survival rate (Zoea Bad), corresponding to the water with the mysis with a good survival rate (Mysis Good), corresponding to the water with the mysis with a bad survival rate (Mysis Bad), and corresponding to the rearing water with the mysis with a low survival rate (Mysis Worst), were constructed. These 6 core microbiomes were ultimately compared together to highlight a core microbiome of the rearing water present throughout the larval development and also specific microbiomes. These core and specific microbiomes of interest were then compared to the water storage microbiomes to decipher the role of the lagoon water on the rearing process.

The LEfSe (80) was used to identify characteristic biomarkers of a specific condition based on their relative abundances and was performed using the microbiomeMarker package (81) in R Studio. LEfSe was calculated using the OTUs common to all rearing water samples to identify biomarkers common to specific conditions. A threshold of 4 was used for the logarithmic LDA score for discriminative features and a *P* value of 0.05 was set for the Kruskal-Wallis tests between the conditions. Then, a correlogram was built between the families identified in the LEfSe, the hydrochemical parameters, and the growth and health status of the larvae present in the rearing water (Nauplii Good, Zoea Good, Zoea Bad, Mysis).

### Data availability.

The data sets generated and analyzed for this study can be found in the NCBI SRA repository under the BioProject ID PRJNA736535 (SRA accession no. SRR17333422 to SRR17333488).
