# Supplementary material for: Microbiota of the Rearing Water of Penaeus stylirostris Larvae Influenced by Lagoon Seawater and Specific Key Microbial Lineages of Larval Stage and Survival
Source: Microbiol Spectr. 2022 Nov 23;10(6):e04241-22. doi: 10.1128/spectrum.04241-22 (PMC9769815; doi:10.1128/spectrum.04241-22)
Supplement: Supplemental file 1 — Table S1. Download spectrum.04241-22-s0001.pdf, PDF file, 0.1 MB [file spectrum.04241-22-s0001.pdf]

Supplementary Table

Supplementary Table 1: Alpha diversity of the water samples.

GR is the primary reservoir sample; RC is the secondary reservoir sample when it was filled with water and C is the secondary reservoir sample after 3 days of water circulating through a skimmer and filters. Control is the water control without larvae, food nor antibiotic; With stands for the rearing water with erythromycin and "Without" for the rearing water without antibiotic.

Sample	OTUs number	Chao1	ACE	Shannon index	Inverse Simpson
GR	426	648	621,94	3,43	9,76
RC	542	741,11	730,99	3,75	11,13
C	457	697,05	677,14	3,83	15,29
Control D0	593	593	593	2,94	5,69
Control D1	524	524	524	3,41	10,96
Control D2	534	534	534	3,33	12,13
Control D3	590	590	590	3,11	9,03
Control D4	666	666	666	3,23	9,31
Control D5	756	756	756	3,49	10,25
Control D6	702	702	702	3,35	10,68
Control D7	731	731	731	3,48	12,18
Control D8	619	619	619	3,38	12,12
Control D9	645	645	645	3,52	13,16
With D1-1	587	587	587	3,65	14,92
With D1-2	537	537	537	2,94	6,08
With D1-3	590	590	590	3,49	12,57
With D2-1	488	488	488	2,93	8,28
With D2-2	451	451	451	2,75	6,40
With D2-3	447	447	447	2,90	8,10
With D3-1	607	877,93	864,29	2,90	7,74
With D3-2	460	693,33	684,85	2,09	3,26
With D3-3	472	696,64	683,14	2,95	8,22
With D4-1	546	778,99	809,19	2,20	2,98
With D4-2	429	596,06	610,99	1,98	3,23
With D4-3	770	1021,73	1008,82	3,29	10,31
With D5-1	655	852,11	861,07	3,54	13,53
With D5-2	573	804,12	807,44	2,31	4,19

With D5-3	624	875,37	879,92	3,38	11,64
With D6-1	763	1053,42	996,99	3,51	11,02
With D6-2	671	905,01	867,61	3,33	10,60
With D6-3	798	964,17	982,84	3,88	21,59
With D7-1	581	764,00	775,92	2,36	4,85
With D7-2	601	812,37	858,23	2,99	10,13
With D7-3	616	809,06	826,49	2,76	5,01
With D8-1	540	762,96	759,31	2,22	4,50
With D8-2	515	678,04	699,57	2,68	7,29
With D8-3	553	813,04	798,86	2,14	3,68
With D9-1	431	654,78	630,76	1,71	3,02
With D9-2	595	799,24	772,15	2,83	7,98
With D9-3	617	891,34	849,62	2,49	4,64
Without D1-1	750	1012,41	1002,46	3,60	13,47
Without D1-2	746	1001,43	1002,19	3,93	22,88
Without D1-3	818	1086,86	1078,34	3,82	19,65
Without D2-1	777	1053,47	1064,10	3,04	6,29
Without D2-2	671	902,89	927,68	3,10	6,10
Without D2-3	610	853,14	838,75	2,90	6,02
Without D3-1	667	885,68	872,25	3,59	13,03
Without D3-2	633	937,94	894,54	3,56	12,62
Without D3-3	608	842,94	869,66	3,49	12,15
Without D4-1	575	748,00	749,83	3,28	8,66
Without D4-2	591	826,69	861,36	3,19	8,91
Without D4-3	611	821,00	837,53	3,40	10,29
Without D5-1	634	899,31	955,93	3,36	7,28
Without D5-2	657	872,10	897,89	3,23	7,81
Without D5-3	566	771,38	812,14	3,42	10,65
Without D6-1	750	963,13	987,36	3,66	14,44
Without D6-2	618	817,16	828,43	4,09	24,10
Without D6-3	698	963,62	922,65	4,15	22,13
Without D7-1	649	917,21	905,70	4,03	25,40
Without D7-2	626	848,08	821,85	3,65	17,58
Without D7-3	633	872,20	878,39	3,44	13,01
Without D8-1	595	782,63	819,03	3,75	15,11
Without D8-2	560	807,92	819,54	3,74	15,94
Without D8-3	489	680,94	712,77	2,99	5,82

Without D9-1	613	890,17	873,19	3,30	7,68
Without D9-2	542	790,18	779,90	3,24	7,33
Without D9-3	512	687,07	695,12	3,01	6,29
